# Single leg drop jump performance identifies functional deficit in collegiate athletes who have returned to sports after ACL reconstruction: A case–control study

**DOI:** 10.1097/MD.0000000000031790

**Published:** 2022-12-09

**Authors:** Han Wei Lem, Shih-Chung Cheng, Hsiao-Yun Chang, Min-Hao Hung, Wen-Ling Yeh

**Affiliations:** a MSc and MPE Dual Programme in International Sport Coaching Science, University of Physical Education, Budapest, Hungary; b MSc and MPE Dual Programme in International Sport Coaching Science, National Taiwan Sport University, Taoyuan, Taiwan; c Graduate Institute of Athletics and Coaching Science, National Taiwan Sport University, Taoyuan, Taiwan; d Department of Athletic Training and Health, National Taiwan Sport University, Taoyuan, Taiwan; e Office of Physical Education, National Chin-Yi University of Technology, Taichung, Taiwan; f Department of Orthopaedic Surgery, Lotung Poh-Ai Hospital, Yilan, Taiwan; g Department of Orthopaedic Surgery, Chang Gung Memorial Hospital, Taoyuan, Taiwan; h College of Medicine, Chang Gung University, Taoyuan, Taiwan.

**Keywords:** jump height, limb asymmetry, mean propulsion force, modified star excursion balance test, reactive strength index, return to sport

## Abstract

Despite its apparent functional importance, there is a general lack of data in explosive strength in individuals recovering from an anterior cruciate ligament reconstruction (ACLR). Hence, we wonder if single leg drop jump (SLDJ) can be an effective testing since drop jump is a commonly used testing which rely on adequate development of explosive strength and stretch shortening cycle function. The purpose of this study was to determine if SLDJ test can identify functional deficit in collegiate athletes who have returned to sports (RTS) after ACLR when comparing it with the common return to sport testing. Nine collegiate athletes who had undergone a unilateral ACLR and returned to their primary sport with at least 10 months post-surgery were recruited and assigned into the injured group and compared with 9 matched non-injured athletes as the control group. Both groups underwent an identical battery testing in 1 session with the sequence of first modified star excursion balance test (SEBT), second single hop and bound test, third SLDJ and lastly 1 repetition maximum (1RM) single leg press. A 2-way mixed model analysis of variance showed that there is no significant interaction effect on common RTS testing which include modified SEBT, single hop and bound tests, and 1RM single leg press, but significant interaction effect on SLDJ jump height (*P* = .03), reactive strength index (*P* = .03) and mean propulsion force (*P* = .03). For the injured group, ACLR leg jump height (10.35 ± 2.71 cm) was significantly lower than non-ACLR leg (12.86 ± 3.51 cm) with a mean difference of 2.51 (95% confidence interval [CI]: 0.55‐4.47). ACLR leg reactive strength index (0.29 ± 0.10 m/s) was significantly < non-ACLR leg (0.39 ± 0.16 m/s) with a mean difference of 0.1 (95% CI: 0.03‐0.17) and ACLR leg mean propulsion force (1087.49 ± 287.26 N) was significantly < non-ACLR leg (1157.40 ± 299.80 N) with a mean difference of 69.91 (95% CI: 16.04 to 123.78). SLDJ was able to identify jump height, reactive strength and propulsion force deficit in the involved limb of collegiate athletes who have returned to sports after ACLR.

## 1. Introduction

Anterior cruciate ligament (ACL) injuries are common in athletes and anterior cruciate ligament reconstruction (ACLR) is a common operation in the management of ACL ruptures. The goals of ACLR in athletes are to restore normal knee joint stability and function to prevent future reinjuries and allow a safe return to preinjury level of sport.^[1]^ However, only 65% returned to their preinjury level of sport and 55% returned to competitive level sport following ACL surgery.^[2]^ Moreover, up to 19% of athletes reinjured the reconstructed ligament, and up to 24% injured the contralateral ACL, with an overall reinjury rate of up to 49% after ACL reconstruction.^[1]^ With the high recurrence rate, there has consequently been marked interest and rapid growth in studies that propose return to sport (RTS) criteria to reduce the risk of a second ACL injury. Typically, these are a set of criteria or “test battery” that is used to clear the athlete for RTS at the final stage of rehabilitation.^[3]^ Whilst the specific content of reported RTS testing batteries has varied, overall, they are designed to incorporate several domains of risk factors. A recent study indicated that an RTS testing battery should at least include a series of times post ACLR, strength tests, hop tests, patient reported outcome measures, clinical examination, and performance-based criteria such as proprioception or balance test.^[4]^

Despite the seemingly low rate of athletes returning to the same pre-injury level of sport and high rate of reinjury, the muscle function tests that are commonly used may not be demanding enough or sensitive enough in detecting functional limitations in ACL-deficient knees.^[5]^ Instead, there is a general lack of data in explosive strength in individuals recovering from an ACLR. In addition to the maximum strength, the indices of explosive strength should also be included in monitoring recovery of knee function following an ACLR.^[6]^ Furthermore, values should be used for the post ACLR side to side comparisons, providing a more valid criterion regarding the muscle recovery and readiness for the RTS. Tests that measure lower limb power output and the reactivity of an athlete might be more useful to assess the physical and performance readiness to RTS.^[7]^ Since RTS decisions are an important component of sports medicine practice and both researchers and clinicians have attempted to develop evidence-based models to facilitate positive outcomes, it is therefore apparent that assessment protocols, consisting of demanding and sensitive tests of neuromuscular function, are required to enable practitioners to make informed RTS decisions.^[8]^ Hence, we wonder if single leg drop jump (SLDJ) can be an effective RTS testing since drop jump is a commonly used plyometric exercise or testing which rely on adequate development of critical motor abilities such as explosive strength and stretch shortening cycle (SSC) function.

The purpose of this study was to determine if the SLDJ test can identify functional deficit in collegiate athletes who have RTS after ACLR when comparing it with the common RTS testing. We hypothesized that the SLDJ would identify deficits in the involved limb of athletes following ACL reconstruction who were cleared for RTS. If this is true, introducing SLDJ as a RTS or return to performance testing for athletes with ACLR may improve the testing batteries sensitivity when evaluating athletes’ lower-extremity function after ACLR. This can better inform clinicians, physiotherapists, athletic trainers, strength and conditioning coaches or any other stakeholders RTS or return to performance decisions, which could in turn limit potential for re-injury and ensure athletes are able to return to performance.

## 2. Methods

### 2.1. Study design and setting

This was a case-control study. The study period was from January 2020 until December 2021. This study undertook a testing battery with collegiate athletes who have RTS after ACLR along with matched control. The research setting was at a sports medicine lab and biomechanics lab in National Taiwan Sport University, Taoyuan, Taiwan.

### 2.2. Participants

Eighteen participants were included in this study. Collegiate athletes who had undergone a unilateral ACLR with an either autologous ipsilateral bone-patellar-tendon-bone or a hamstrings graft (semitendinosus or semimembranosus) and returned to their primary sport with at least 10 months and a maximum of 30 months following reconstruction were recruited to participate and allocated into the injured group (n* *= 9). Participants in the injured group were asked to bring 1 teammate with them to serve as control group participants (n* *= 9) matched for sport, activity level, gender, height, weight, and age.^[9]^ The athletes who were included must be collegiate athlete with an age of at least 20 years old with no other or subsequent injuries to the ACL reconstructed limb, no injury to the uninvolved knee, hips, ankles or back. Subjects should have no pain, swelling or decreased range of motion in clinical knee-joint examination, performed on both athletes’ knees by an experienced physiotherapist or athletic trainer. In addition, their Tegner Activity Scale should score at least 7 and international knee documentation committee (IKDC) Subjective Knee Evaluation Form should have a score of at least 70.^[10]^ The legs of the injured group athletes were classified as ACLR leg or non-ACLR leg while the legs of the control group athletes were classified as dominant leg or non-dominant leg. Leg dominance was determined based on a questionnaire on the preferred limb to jump.^[11]^ Legs of the participants were matched as stronger leg or weaker leg for further data analysis. Stronger leg was defined as non-ACLR leg in the injured group or dominant leg in the control group while weaker leg was defined as non-ACLR leg in the injured group or non-dominant leg in the control group. This study was approved by the Institutional Review Board of the Fu Jen Catholic University (ref: C108049) and informed consent was obtained from all participants prior to data collection.

### 2.3. Procedure

Participants were asked to wear sports clothing and their own athletic shoes. Each participant had to perform a series of battery testing in 1 session with the sequence of first modified star excursion balance test (SEBT), second single hop and bound test, third SLDJ and lastly 1 repetition maximum (1RM) single leg press. Before the testing battery, athletes started with a standard 15 to 20 minutes warm up of static bicycle cycling and dynamic stretching. The order of limb testing started with the stronger leg followed by the weaker leg. Each participant performed 3 practice trials and 3 successful trials for each of the different tests. The 3 successful data collected in all variables of interest was calculated into mean. All participants were evaluated at the same location by the same examiner.

#### 2.3.1. *Modified star excursion balance test*.

Due to task redundancy and the time consumed while performing in all 8 reach directions, a modified version of the SEBT has been recommended. The modified SEBT uses only 3 directions which is anterior, posteromedial, and posterolateral. The modified SEBT is a functional screening tool to assess lower extremity dynamic stability and neuromuscular control, encompassing lower extremity strength, coordination, balance, and flexibility.^[12]^ A good to excellent relative within-session intra-rater reliability between the 3 trials on specified directions was reported in a previous study, with an intraclass correlation coefficients (ICC) from 0.90 to 0.95.^[13]^ Prior to testing, leg length was measured from the most distal aspect of the anterior superior iliac spine to the most distal aspect of the medial malleolus for each leg.^[14]^ The participants were instructed to stand in single-limb stance on the test limb with the most distal aspect of their great toe at the center of a modified SEBT grid without wearing shoes. Participants were instructed to perform maximal reaches with the non-stance limb in the sequence of anterior, posteromedial, and posterolateral directions, while maintaining single-leg stance on the test limb. Trials were discarded and repeated if the individual was unable to maintain a single-limb stance throughout the test, the hands off from the hips during the test, the heel of the stance foot did not maintain contact with the floor throughout the test, weight was shifted onto the reach foot, or the reaching limb did not return to the start position prior to another trial.^[12]^ Composite reach distance score was calculated as the sum of the mean anterior, posteromedial, and posterolateral reach distances divided by 3 times leg length and multiplied by 100.^[12]^

#### 2.3.2. *Single hop and bound tests.*

In a recent review, the authors suggested the use of more than 2 hop tests does not appear to be necessary due to high collinearity and no greater sensitivity to detect abnormality.^[15]^ However, inclusion of other jump tests in different planes to give greater information about the current function of the knee may be needed.^[15]^ Hence, in our study, the tasks used were the anterior hop, medial side bound, and lateral side bound. Reliability of the hop tests has been reported to be excellent, with an ICC ranging from 0.92 to 0.96.^[16]^ When performing the 3 trials for each task, the participants were instructed to stand on tested lower extremity and to position the toes on a mark on the floor. The distance was measured from the toe in the starting position to the heel where the athlete landed. For anterior hop, participants were instructed to jump forward as far as possible and land on the same leg. For medial side bound, participants were instructed to jump to medial side as far as possible and land on another leg. For lateral side bound, participants were instructed to jump to lateral side as far as possible with another leg crossover and land on the crossover leg. A trial was deemed successful if the participant landed on 1 foot and maintained the landing position without losing balance for at least 2 seconds, no extra hops for balance correction were allowed and until the investigator marked on the floor where the participant landed. The single anterior hop was performed first, followed by the medial side bound and the lateral side bound. A 2-minutes rest between trials was allowed.

#### 2.3.3. *Single leg drop jump*.

All the participants held their hands on their waist and performed the SLDJ by stepping off a 20 centimeters platform onto a force plate (Kistler type 9287A, Winterthur, Switzerland) with a frequency of 2000 Hz connected directly to a computer with NEXUS 1.8.5 software to acquire data. A trial was deemed successful if the participant landed on 1 leg and maintained the landing position without losing balance for at least 2 seconds, no extra hops for balance correction and no hands off from the hips during the test. As a previous study, we stressed consistently between trials that participants should minimize their ground contact time and jump as high as possible by verbal instruction.^[17]^ A 2-minutes rest between trials was allowed. Test-retest reliability of SLGJ has been reported to be good to excellent, with an ICC ranging from 0.75 to 0.96.^[18]^ Self-written MATLAB 2015b program was used for data processing. The force plate data was filtered by using Butterworth 4^th^-Order low-filtered of 40 Hz. Vertical ground reaction force of 50 Newtons was used to detect the moment of landing. Table 1 showed the SLDJ variables description and calculation method used in the self-written MATLAB program.^[19,20]^

**Table 1:** SLDJ variables description and calculation method.

Variables	Unit	Variables description/Calculation method
CT	s	Ground contact end time—ground contact start time
Ground contact start time	s	vGRF > 10 N
Ground contact end time	s	vGRF < 10 N
FT	s	Flight end time—flight start time
Flight start time	s	vGRF < 10 N
Flight end time	s	vGRF > 10 N
JH	m	JH = FT^2^ × 9.81/8
RSI	m/s	Jump height/contact time
Mean braking force	N	The average force produced in the braking phase
Peak braking force	N	The maximum force produced in the braking phase
Braking phase	s	One sample after the instant of peak negative COM velocity and continues through to zero COM velocity
Mean propulsion force	N	The average force produced in the propulsion phase
Peak propulsion force	N	The maximum force produced in the propulsion phase
Propulsion phase	s	This phase begins when a positive COM velocity is achieved (one sample after zero) until take-off (a force threshold equal to 5 times the standard deviation of flight force when the force platform is unloaded)

COM = center of mass, CT = contact time, FT = flight time, JH = jump height, RSI = reactive strength index, SLDJ = single leg drop jump.

#### 2.3.4. *1RM single leg press*.

The 1 repetition maximum (1RM) leg press strength test was performed using a horizontal leg press training machine (Matrix G3-S70, Wisconsin). A previous study reported a good intra-rater, test-retest reliability with ICC of 0.94 to 0.98 showed 1RM leg press is a reliable measurement to assess muscle strength.^[21]^ The athletes were instructed to perform the single leg press at 90º of knee flexion measured by using a goniometer. The 1RM testing protocol used in the study as described by Sheppard and Triplett.^[22]^ Verbal encouragement was provided during the 1RM approach. For the 1RM single leg press, relative result was calculated as follow: (Absolute data of 1RM [kg] ÷ body weight obtained from force plate [kg]) × 100.

### 2.4. *Statistical analyses*

Means and standard deviations were calculated for all variables of interest. Statistical analysis was conducted in Statistical Package for the Social Sciences Version 25 (SPSS Inc, Chicago, IL). Independent sample *t*-test and Fisher’s Exact test were used to analyze any heterogeneity between group. To determine differences between legs (stronger and weaker) and groups (injured and control), a 2-ways mixed model of analysis of variance (ANOVA) tests was used to determine the interactions and main effects for each of the battery testing measures. Statistical significance was set at an alpha level of.05. Partial eta squared ($\eta_{P}^{2}$) was reported as the effect size of ANOVA test with.01 ≤ $\eta_{P}^{2}$ <.06 was a small effect, .06 ≤ $\eta_{P}^{2}$ <.14 was a medium effect, and.14 ≤ $\eta_{P}^{2}$ was a large effect.^[23]^ A power analysis (G* Power, Version 3.1.9) with ANOVA repeated measures, between factors was used to calculate the required sample size. With an effect size *f* of.59 (determine from $\;\eta_{P}^{2}$ of interaction effect on SLDJ jump height), an alpha level of *P* ≤ .05, number of measurements of 4, total sample size of 18 was required to obtain power of 0.8.

## 3. Results

The demographic and functional characteristics of the injured group and control group were shown in Table 2. All the variables do not show significant difference except the IKDC subjective knee evaluation form score (*t *= -3.07, *P = *.01). Injured group IKDC score (83.53 ± 10.07) was significantly lower than control group IKDC score (94.89 ± 4.67).

**Table 2:** Demographic and functional characteristics of research participants.

	Injured (n = 9)	Control (n = 9)	*P*
Male (count, %)	3 (33.33%)	3 (33.33%)	1.000$
Age (yr)#	21.78 ± 2.99	21.00 ± 1.41	.491
Height (m)#	1.70 ± 0.16	1.71 ± 0.12	.935
Weight (kg)#	66.75 ± 15.47	68.24 ± 18.11	.854
Body mass index (kg/m²)	22.76 ± 2.90	22.94 ± 3.26	.901
Sport (count, %)			1.000$
Basketball	3 (33.33%)	3 (33.33%)	
Soccer	3 (33.33%)	3 (33.33%)	
Judo	2 (22.22%)	2 (22.22%)	
Volleyball	1 (11.11%)	1 (11.11%)	
Weaker leg (ACLR/non-dominant leg)	4 Left/ 5 Right	4 Left/ 5 Right	1.000$
Tegner activity scale#	8.11 ± 1.05	8.11 ± 1.05	1.000$
IKDC subjective knee# evaluation form score#	83.53 ± 10.07	94.89 ± 4.67	.010*
ACLR graft			
Semitendinosus	7		
Semimembranosus	1		
Bone patellar tendon bone	1		
Time post ACLR (days)#	697.00 ± 234.36		

ACLR = Anterior Cruciate Ligament Reconstruction, IKDC = International Knee Documentation Committee.

$Obtained from Fisher’s exact test.

#Mean ± SD, obtained from independent sample *t*-test.

* *P* < .05.

Results showed that there is no significant interaction effect of groups and legs in all common RTS battery testing which include the modified SEBT test, single hop and bound tests, and 1RM single leg press test as shown in Table 3.

**Table 3:** Comparison of modified star excursion balance test, hop tests, and 1RM single leg press variables.

Common RTS testing variables	Injured group (n = 9)			Control group (n = 9)			Legs × groups interaction effect
	Non-ACLR leg	ACLR leg	Mean difference (95% CI)	Dominant leg	Non-dominant leg	Mean difference (95% CI)	
	(mean ± standard deviation)			(mean ± standard deviation)			*P*
Modified SEBT anterior reach distance (cm)	62.52 ± 8.80	64.44 ± 11.35	–1.92 (–5.72 to 1.88)	65.11 ± 10.98	66.05 ± 11.23	–0.94 (–3.69 to 1.81)	.64
Modified SEBT posteromedial reach distance (cm)	98.45 ± 33.49	97.25 ± 30.79	1.20 (–2.55 to 4.95)	92.27 ± 35.78	93.80 ± 37.02	–0.53 (–5.34 to 4.27)	.64
Modified SEBT posterolateral reach distance (cm)	97.03 ± 35.48	98.27 ± 37.69	–1.24 (–5.24 to 2.77)	91.30 ± 36.69	94.16 ± 40.31	–2.87 (–9.20 to 3.47)	.62
Modified SEBT composite score (%)	98.44 ± 27.75	99.29 ± 28.49	–0.85 (–2.58 to 0.88)	93.22 ± 30.35	94.04 ± 30.93	–0.82 (–4.70 to 3.06)	.99
Anterior single hop distance (cm)	127.56 ± 18.37	123.78 ± 19.47	3.79 (–5.69 to 13.26)	135.70 ± 23.18	134.22 ± 21.85	1.49 (–3.63 to 6.60)	.63
Medial single bound distance (cm)	150.44 ± 16.09	149.49 ± 18.99	0.95 (–2.65 to 4.55)	153.86 ± 16.86	152.30 ± 16.00	1.56 (–3.06 to 6.18)	.81
Lateral single bound distance (cm)	118.43 ± 21.50	114.92 ± 23.78	3.51 (–2.49 to 9.51)	107.41 ± 18.35	109.84 ± 18.62	–2.43 (–7.56 to 2.70)	.10
Single leg press 1RM (kg)	59.28 ± 30.19	58.83 ± 27.84	0.44 (–4.37 to 5.25)	63.63 ± 32.95	61.48 ± 30.81	2.16 (–2.64 to 6.95)	.57
Relative single leg press (% of body weight)	86.66 ± 25.71	86.48 ± 22.50	0.17 (–8.39 to 8.74)	90.19 ± 24.56	88.01 ± 25.43	2.17 (–5.31 to 9.65)	.69

1RM = one repetition maximum ACLR = anterior cruciate ligament reconstruction, CI = confidence interval, RTS = return to sport, SEBT = star excursion balance test.

However, results showed that there was significant interaction effect of groups and legs in the SLDJ jump height (F_1,16_ = 5.61, *P* = .031, $\;\eta_{P}^{2}$ =.26), reactive strength index (RSI) (F_1,16_ = 5.95, *P* = .027, $\;\eta_{P}^{2}$ =.27), and mean propulsion force, (F_1,16_ = 5.55, *P* = .03, $\;\eta_{P}^{2}$ =.26) as shown in Table 4. There was no significant interaction effect in the SLDJ contact time (F_1,16_ = 1.13, *P* = .303), mean braking force (F_1,16_ = 3.60, *P* = .076), peak braking force (F_1,16_ = 2.56, *P* = .13) and peak propulsion force, *F* (1, 16) = 3.92, *P* = .065). For the injured group SLDJ jump height, leg had a significant simple main effect (*F*_1,16_ = 14.71, *P* = .002, $\;\eta_{P}^{2}$ =.48) which ACLR leg jump height (10.35 ± 2.71 cm) was significantly < non-ACLR leg (12.86 ± 3.51 cm) with a mean difference of 2.51 (95% confidence interval [CI]: 0.55‐4.47). For the control group SLDJ height, leg does not have a significant simple main effect (*F*_1,16_ = 0.24, *P* = .63). For the injured group SLDJ RSI, leg had a significant simple main effect (F_1,16_ = 15.00, *P* = .001, $\;\eta_{P}^{2}$ =.48) which ACLR leg RSI (0.29 ± 0.11 m/s) were significantly < non-ACLR leg (0.39 ± 0.16 m/s) with a mean difference of 0.1 (95% CI: 0.03‐0.17). For the control group SLDJ RSI, leg does not have a significant simple main effect (*F*_1,16_ = 0.12, *P* = .73). For the injured group SLDJ mean propulsion force, leg had a significant simple main effect (*F*_1,16_ = 9.89, *P* = .006, $\;\eta_{P}^{2}$ =.382) which ACLR leg mean propulsion force (1087.49 ± 287.26 N) was significantly < non-ACLR leg (1157.40 ± 299.80 N) with a mean difference of 69.91 (95% CI: 16.04‐123.78). For the control group SLDJ mean propulsion force, leg does not have a significant simple main effect (*F*_1,16_ = 0.03, *P* = .86).

**Table 4:** Comparison of SLDJ variables.

Single leg drop vertical jump variables	Injured group (n = 9)			Control group (n = 9)			Legs × groups interaction effect		Simple main effect		
	Non-ACLR leg	ACLR leg	Mean difference (95% CI)	Dominant leg	Non-dominant leg	Mean difference (95% CI)			Non-ACLR and ACLR Leg		Dominant and non-dominant leg
	Mean ± standard deviation			Mean ± standard deviation			*P*	$\;\eta_{P}^{2}$	*P*	$\eta_{P}^{2}$	*P*
Jump height (cm)	12.86 ± 3.51	10.35 ± 2.71	2.51 (0.55‐4.47)	12.36 ± 3.57	12.04 ± 3.78	0.32 (–0.53 to 1.17)	.031*	.260	.002**	.479	.632
Contact time (s)	0.35 ± 0.09	0.37 ± 0.07	–0.02 (–0.04 to 0.01)	0.37 ± 0.09	0.36 ± 0.06	0.01 (–0.03 to 0.04)	.303				
Reactive strength index (m/s)	0.39 ± 0.16	0.29 ± 0.10	0.1 (0.03‐0.17)	0.35 ± 0.11	0.34 ± 0.11	0.01 (–0.4 to 0.06)	.027*	.271	.001**	.484	.732
Mean braking force (N)	1375.63 ± 308.37	1244.27 ± 236.81	131.35 (42.82‐219.88)	1282.39 ± 321.11	1266.52 ± 348.48	15.87 (–92.96 to 124.70)	.076				
Peak braking force (N)	1992.02 ± 467.27	1856.13 ± 421.57	135.89 (27.27‐244.52)	1787.82 ± 498.87	1802.90 ± 531.67	–15.08 (–203.89 to 173.72)	.130				
Mean propulsion force (N)	1157.40 ± 299.80	1087.49 ± 287.26	69.91 (16.04 to 123.78)	1153.84 ± 341.90	1157.97 ± 363.21	–4.13 (–52.66 to 44.40)	.032*	.257	.006**	.382	.855
Peak propulsion force (N)	1824.76 ± 573.36	1682.03 ± 503.98	142.73 (15.04‐270.42)	1680.78 ± 468.44	1715.89 ± 563.58	–35.11 (–198.14 to 127.92)	.065				

ACLR = anterior cruciate ligament reconstruction, CI = confidence interval, SLDJ = single leg drop jump.

* *P* < .05.

** *P* < .01.

## 4. Discussion

Apart from time after ACLR should be at least 9 months, no pain, no effusion, normal range of motion, and IKDC subjective knee evaluation form should have a score of at least 70, which was set as the research participants’ inclusion criteria. There is not any significant difference between groups nor within group in other common RTS criteria, which include the dynamic balance, single leg strength, and hop tests. Limb symmetry indexes (LSI) use concurrent measures of the uninvolved limb as a reference with a standard of LSI ≥ 90% are often used as a criterion for RTS.^[5]^ However, bilateral muscle strength and hop performance deficits have been demonstrated after ACLR.^[15,24,25]^ These studies found LSI frequently overestimate athletes’ knee function after ACLR and raise concern about whether the RTS criteria by using LSI utilized in current clinical practice after ACLR are stringent enough to achieve a safe and successful RTS. Hence, without present the RTS performance testing in LSI, we present the absolute value and compare it to the match leg of the control group.

For the balance test, there was no significant difference between groups nor within group in modified SEBT in all 3 different directions (anterior, posteromedial, and posterolateral) and composite score in collegiate athletes who had RTS after ACLR. Our findings were supported by a previous study which found that performance deficit in the modified SEBT was not maintained after ACLR and rehabilitation.^[26]^ In addition, previous works had reported no between-leg differences in dynamic postural stability in individuals who had undergone ACLR at an average of 18 months post-surgery.^[27]^ We agree with Domingues et al and Mattacola et al that sensorimotor training and strengthening of lower limbs oriented from the early stages of ACLR rehabilitation can help to restore neuromuscular control and further eliminate dynamic balance deficit.^[26,27]^

Knee extensor strength is of particular interest after ACLR, as it is well-recognized to be impaired in the ACLR knee and more strongly associated than knee flexor strength with future knee function and activity levels.^[28]^ Strength deficit after injury is linked to poor biomechanics, reduced knee function, increased knee osteoarthritis risk, as well as heightened risk of re-injury upon RTS.^[29]^ For the single leg strength, there was no significant difference between groups nor within group in single leg press either in absolute or relative 1RM. A previous study has also shown a similar result, in which 45 male professional soccer players demonstrated no significant difference in isometric leg-press strength between involved and non-involved leg either in 6 months post ACLR or 12 months post ACLR.^[30]^ The restoration of knee extensor muscle strength represents a mid-stage rehabilitation marker, 1 which should be achieved prior to restoring movement quality, functional strength, power, and explosive muscle strength as well as subsequent sport-specific re-training and RTS.^[29]^ In addition, another study also showed similar quadriceps strength between the ACLR and non-ACLR leg in 83 athletes after perturbation training and aggressive quadriceps strengthening 6 months post reconstruction.^[31]^ In contrast to our findings, 1 study found there was significant difference between the ACLR leg and non-ACLR leg of the patients who underwent ACLR in single leg press muscular power at all pre-operation, 6, 12, and 24 months post operation.^[32]^ In the study, the authors stated that difficulty in restoring strength after an ACL injury could be due to insufficient rehabilitation protocols, rather than neuromuscular deficit.^[32]^ While many factors influence strength after ACLR, pre- and post-operative rehabilitation remain important. A previous study also showed strength asymmetries can be reduced, and normal limb symmetry can be restored through a rehabilitation program consisting of perturbation training and aggressive quadriceps strength preoperatively and a systematic criteria-based program post-operatively.^[31]^ In our study, the participants were all collegiate athletes who may be more diligent in their rehabilitation and have a well-planned rehabilitation protocols which focus on both sport-specific exercises and weight training with optimal loading that aim to prepare the athletes to RTS.

For the single hop and bound tests performance, there was no significant difference between groups nor within group in all 3 tests with different directions. A previous study also showed similar results in which 29 high school and collegiate athletes who underwent ACLR had no significant difference between the involved and uninvolved limbs in single hop for distance.^[33]^ In contrast to our findings, many studies found absolute scores for the non-ACLR leg were significantly better than the ACLR limb for the single hop for distance or side hop at a range of 6 to 24 months post-surgery.^[9,25,32,34]^ Previous studies had shown that strength after ACLR was significantly associated with hop distance. This may explain why our findings were different than other studies as our participants’ single leg strength did not show asymmetry, this may associate with our participant’s hop and bound test performance.^[28,33,35–37]^

Many reviews have shown the common clinical criteria to determine readiness for RTS typically rely on a strength test and a hop test battery to assess functional leg symmetry.^[1,4,38]^ However, more and more researchers or practitioners have mentioned that the common strength and a hop test battery was insufficient to detect deficits in knee function after anterior cruciate ligament reconstruction (ACLR).^[1,5,38]^ Our study results agreed with these researchers as there is no significant interaction between groups and legs in the single leg strength and hop test performance but significant interaction effect in SLDJ variables. Specifically, there was significant lower jump height (JH), reactive strength index (RSI) and mean propulsion force (MPF) in the ACLR leg compared to non-ACLR leg in the injured group while no significant difference in the control group. Two recent studies had a similar result with this study’s findings where there were significant differences between the involved and non-involved legs in JH and RSI during the SLDJ was shown in athletes after ACLR at the time to RTS and also athletes with knee injury in the final phase of rehabilitation prior RTS.^[7,8]^

A previous study has shown a significant high correlation between jump height and RSI as well as a high correlation between RSI and concentric force production which can explain these 3 variables were inter-corelated.^[39]^ Hence, the significant lower jump height and mean propulsion force in injured group athlete’s ACLR leg was due to functional deficits in reactive strength capabilities in the propulsion phase which the athletes need to produce force rapidly from eccentric to concentric work.^[40]^ The RSI asymmetry in the injured group may be due to inhibition in the SSC as RSI has been used in the practical strength and conditioning setting as well as in the exercise science literature to quantify SSC performance.^[41,42]^ Besides, this may also be due to neuromuscular control and the short latency component of the stretch reflex and protective inhibition.^[43]^ Altered neuromuscular control at the hip and knee during a drop jump has been prospectively linked with the incidence of primary as well as secondary ACL injury in athletes.^[44,45]^ A recent study also show that athletes with significant asymmetries in SLDJ performance exhibited differences in knee work and kinematic and altered muscle coordination strategies between involved leg and non-involved leg after ACLR.^[7]^ Asymmetries in limb performance during athletic tasks may be potential risk factors for lower extremity injury, particularly second ACL injury, and should be minimized prior to RTS following ACL reconstruction.^[9]^ A previous study also found jump height and RSI asymmetry during the SLDJ were significantly correlated with 10 minutes, 30 minutes, and 505 change of direction (COD) performances, indicating that larger asymmetries are indicative of slower sprint and COD times.^[46]^ Indeed, it has been suggested that the ability to attenuate, regenerate, and redirect forces acting on a single limb are likely relevant in an injury-risk.^[9]^

The data presented here indicate substantial deficits in jump height, reactive strength, and propulsion force can inform rehabilitation programs for athletes in the late stages of their RTS or return to performance progression. Specifically, exercises designed to elicit rapid responses from the neuromuscular system are likely to enhance rehabilitative outcomes. Exercises designed to invoke rapid motor unit activation will likely prove beneficial in decreasing the functional deficits observed in reactive strength and propulsion force. The rehabilitation program should incorporate a combination of strength and plyometric exercises in the later phase of rehabilitation. Previous literature has suggested that reducing limb asymmetry may be best achieved using unilateral training over bilateral methods.^[47]^

The strength of this study is that it is 1 of the first to apply the SLDJ in athletes with ACLR and compare it with the matched control. In addition, we compare this SLDJ with the common RTS testing and show that the SLDJ were able to determine functional deficit in athletes with ACLR, specifically in reactive strength which can inform rehabilitation programs for athletes in the late stages of their RTS progression.^[8]^ The SLDJ test has the potential to provide important information on neuromuscular deficits that may compromise return to performance by challenging the reactive capacity of the neuromuscular system during the rapid transition from eccentric to concentric work.^[8]^ Our study results also show that SLDJ was a useful test for detecting existent between-limb asymmetry, which agree with Bishop et al^[46]^ SLDJ is easy to administer and could be widely adopted in practical settings given the ability of phone applications to assess jump height, contact time and reactive strength index in drop jumps accurately and reliably when compared with a force plate measuring at 1200 Hz.^[7,8,48]^ The limitations of this study were the surgical methodology or graft type was not controlled, which could potentially affect our results while detailed surgery data such as graft fixation, graft components, and bundle configuration were not considered in the current study. In addition, we were unable to control the rehabilitation protocol of the athletes prior and after ACLR which could potentially affect the testing results. We also lack pre-injury testing data to compare with the data collected. Since the study was conducted only on collegiate athletes, the results may not generalize to the entire population. Future studies should further elaborate on these findings on a larger scale and compare the testing performance data with the pre-injury testing data.

## 5. Conclusion

SLDG identified jump height, reactive strength and propulsion force deficit persist in the involved limb of collegiate athletes who have RTS after anterior cruciate ligament reconstruction. This provides justification for the use of a SLDJ test to better inform clinicians, physiotherapists, athletic trainers, strength and conditioning coaches, and any other stakeholders’ RTS or return to performance decisions, which could in turn limit potential for re-injury and ensure athletes are able to return to performance. While the SLDJ may not identify the exact nature of dysfunction, the task may be sufficiently demanding to provide a global picture of overall function to inform rapid force production and reactive strength capabilities for athletes with anterior cruciate ligament reconstruction.

## Author contributions

**Conceptualization:** Han Wei Lem.

**Data curation:** Shih-Chung Cheng, Wen-Ling Yeh.

**Formal analysis:** Shih-Chung Cheng, Min-Hao Hung.

**Funding acquisition:** Wen-Ling Yeh.

**Investigation:** Han Wei Lem, Hsiao-Yun Chang.

**Methodology:** Han Wei Lem, Hsiao-Yun Chang.

**Supervision:** Shih-Chung Cheng, Wen-Ling Yeh.

**Writing – original draft:** Han Wei Lem.

**Writing – review & editing:** Shih-Chung Cheng, Wen-Ling Yeh.
